# A systematic description of continent ileostomy revision techniques

**DOI:** 10.1007/s00384-022-04282-9

**Published:** 2022-11-21

**Authors:** Nils Karl Josef Ecker, Mathias Tönsmann, Karl-Wilhelm Ecker

**Affiliations:** 1 Lübeck, Germany; 2 Hagen, Germany; 3 Dept. of General, Visceral, and Pediatric Surgery, University of Saarland, Homburg, Saarland Germany; 4 Surgical Dept, MediClin Müritz-Klinikum, Weinbergstraße 19, 17192 Waren, Germany; 5 Tangstedt, Germany

**Keywords:** Nipple valve, Pouch fistula, Stoma complication, Crohn’s disease, Surgical repair, Technical details

## Abstract

**Purpose:**

Comprehensive description of surgical techniques for revision of complications of continent ileostomy (CI).

**Methods:**

By analyzing 133 revision procedures performed over 30 years, a systematically classified approach to the appropriate techniques for CI revision surgery has been derived. Based on the anatomic site and severity of the respective complication, four classes of revision surgeries have been defined: class 1 refers to the nipple valve, class 2 to the pouch, class 3 to the stoma, and class 4 to the afferent loop. The severity of the complication or the complexity of the revision procedure is indicated by a subdivision from a to d.

**Results:**

The surgical variants (class 1a–d, class 2a–c, class 3a–b, and class 4a–b) are shown in schematic illustrations with accompanying descriptions of technical details, the respective fields of application, and the special indications.

**Conclusion:**

Based on these classes of revision surgeries, the specialized surgeon may find differentiated techniques at their disposal to save the CI and avoid unnecessary sacrifice of the artificial continence organ.

## Introduction

Continent ileostomy (CI) is a technical refinement of the conventional ileostomy (IS) that provides voluntary control over fecal evacuation. Due to the absence of an external bag, the quality of life with CI is significantly improved [1, 2]. An intraabdominal low-pressure reservoir (the pouch) and a continence mechanism (the nipple valve (NV)) are prerequisites for proper function [3]. The method was introduced in 1969 by Prof. Nils Kock of Gothenburg [4], leading to the common designation “Kock pouch” (KP).

Apart from the fact that CI has now been replaced by ileal pouch–anal anastomosis (IPAA) as the procedure of choice for proctocolectomy [5], manifold complications also stand in the way of widespread CI use [1, 6]. However, there are numerous patients who received CI or KP decades ago. Furthermore, patients remain today who are candidates for CI but not suitable for IPAA [2]. In addition, there is an increasing number of patients who eventually experience functional failure after temporarily good IPAA function, thus representing an indication for conversion to CI [7]. All these individuals may be at risk of developing late complications of CI. This results in a need for revision surgery.

The senior author has over 3 decades of experience with CI revision and corrective surgery. Since reports on this topic are extremely sparse, an update seems appropriate. The clinical data, including the results of revision surgeries, have been reported recently [8]. Herein, it is exclusively intended to present a systematic description of the surgical techniques based on the characteristics of the respective complications.

## Methods

The medical records of 77 patients who had undergone a total of 133 CI revision surgeries between 1986 and 2015 were reviewed. Revision procedures were classified according to the anatomic site of the underlying complication:

- Class 1: revision surgery of the nipple valve
- Class 2: revision surgery of the pouch
- Class 3: revision surgery of the stoma
- Class 4: revision surgery of the afferent loop

Subtypes were specified according to the clinical severity of the complication and complexity of the surgical repair:

- a: minor clinical severity/easy surgical correctability
- b–d: increasing severity/more difficult correction

The characteristic features of the complications were summarized, and schematic illustrations were prepared for the appropriate surgical procedures. A primary S-design of the pouch was taken as the basis.

## Results

### Nipple valve revision surgery (class 1)

#### Class 1a: incipient slippage with/without detachment of the pouch from the abdominal wall

**Fig. 1:**
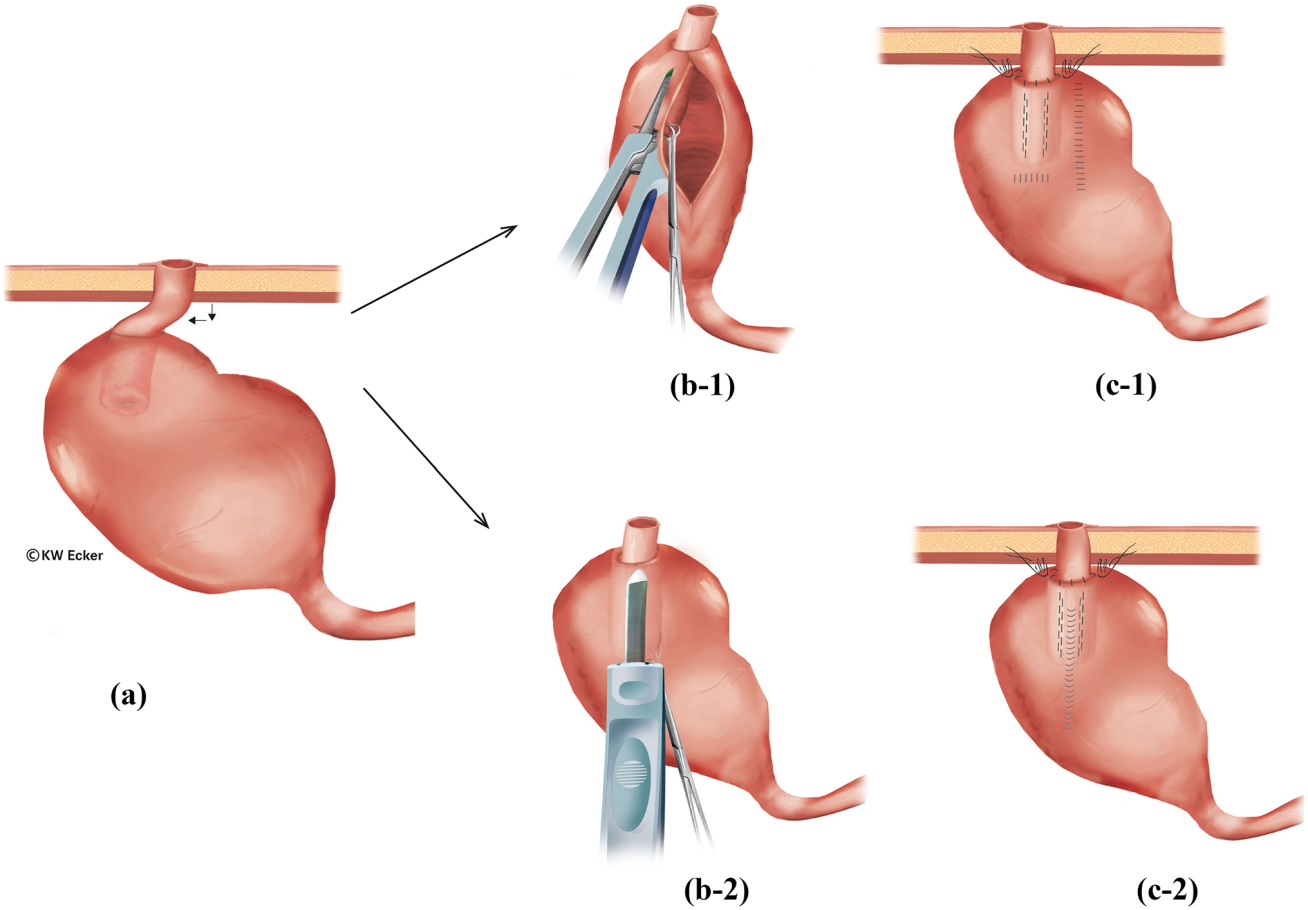
**Restabilization of the nipple valve (NV) in case of pouch detachment from the abdominal wall and incipient valve sliding (class 1a)**. (a) The pouch has detached from the abdominal wall, resulting in a bayonet-like displacement of the outlet duct and shortening of the valve. This can result in both difficult intubation and in incontinence. There are two options for correction. (b-1) According to the first option, the pouch is incised longitudinally at the anterior wall to re-stretch the NV under traction with a Babcock clamp. Via an additional transverse incision of the pouch, fixation of the NV in a maximally stretched position against the anterior wall is performed using a stapler or bladeless linear cutter. Both cuff layers of the NV and the pouch wall are grasped. (c-1) The pouch incisions are closed by suturing, and the pouch is repositioned with stable attachment to the abdominal wall.. (b-2) According to the second option, only a small longitudinal incision is made on the pouch anterior wall, over which the NV is stretched and fixed to the anterior wall as in the first method.(c-2) The pouch incision is closed by suturing, and the pouch is repositioned with stable attachment to the abdominal wall

Incipient slippage is caused by traction of the mesentery on the tip of the NV leading to shortening of the nipple. It is often triggered by detachment of the pouch from the abdominal wall. This complication becomes symptomatic either because intubation is more difficult as a result of bayonet-like deformation of the outlet, or because full continence has been lost as a result of NV shortening. A revision indication is given according to the severity of the disturbance (Fig. 1).

#### Class 1b: pronounced slippage (complete de-intussusception) and prolapse

**Fig. 2:**
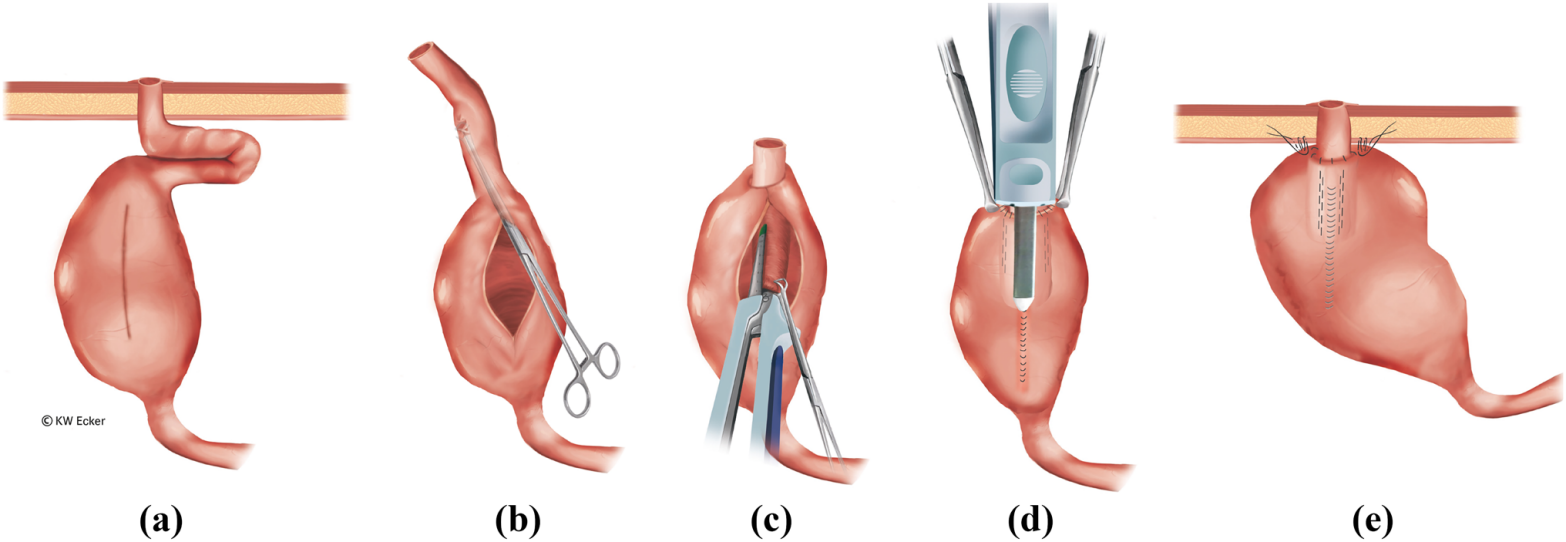
**Restabilization of the nipple valve (NV) in case of complete de-intussusception of the NV (class 1b)**. (a) The pouch is widely detached from the abdominal wall, and the NV is completely de-intussuscepted. (b) After complete removal of the pouch, it is incised longitudinally at the anterior wall. A Babcock clamp is used to grasp the efferent loop in order to re-intussuscept the loop. (c) As with an original construction, the NV is stabilized on both sides of the irradiating mesentery with one staple application on each side (four rows) using the bladeless linear cutter. (d) After the anterior wall has been reclosed by suturing, the outer cuff of the NV is stapled over this suture against the pouch anterior wall using the bladeless linear cutter. (e): Interrupted sutures are still placed in the area of the pouch shoulder to secure the telescope, and the pouch is repositioned stably against the abdominal wall by interrupted sutures

The end result of an unhindered sliding process is ultimately complete de-intussusception of the NV. Complete incontinence then represents an absolute indication for re-intussusception and restabilization (Fig. 2). A similar situation exists with prolapse of the valve through the stoma to the outside.

#### Class 1c: destruction and/or irreparability of the nipple valve

**Fig. 3:**
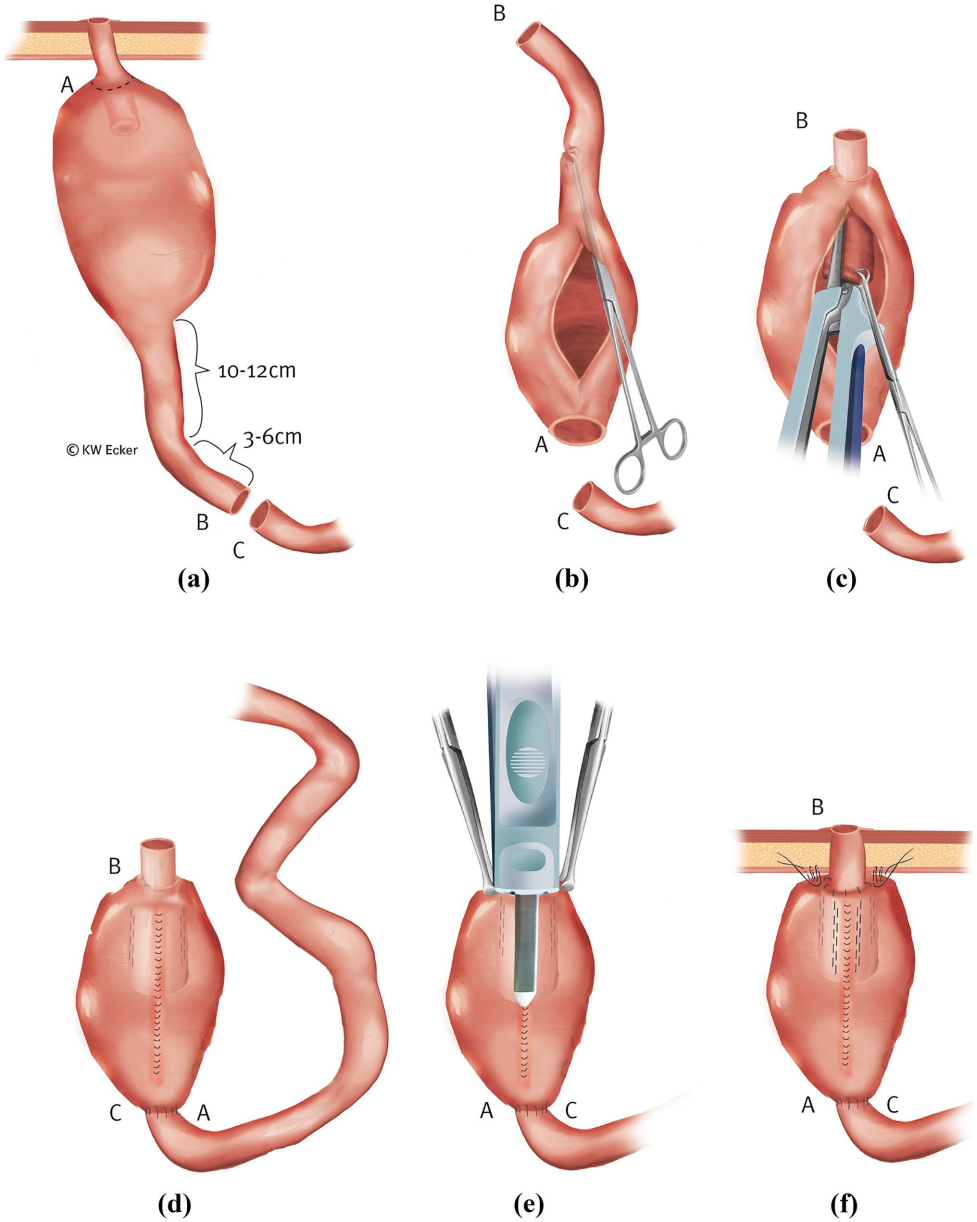
**Reconstruction of the nipple valve (NV) from the afferent loop (class 1c)**. (a) In the case of a destroyed NV, i.e., one that can no longer be restabilized, but an afferent loop suitable for valve formation, 10–12 cm of this is marked for a new NV and 3–6 cm for the outlet duct and stoma. (b) After complete mobilization of the pouch, the former NV is resected; the afferent loop is transected between B and C, 13 and 18 cm orally of the pouch; and the pouch is rotated by 180°. Through a longitudinal incision on the anterior wall, a Babcock clamp is inserted into the new outlet duct (the former afferent loop) to initiate intussusception. (c) As with an original construction, the NV is stabilized on both sides of the irradiating mesentery with one application on each side (four rows) of the bladeless cutter device. (d) The anterior wall of the pouch is closed by suture, and intestinal continuity is restored by enteroanastomosis (C–A). (e) Across the front wall suture, the outer cuff of the NV is stapled against the pouch wall with the bladeless linear cutter. (f) Interrupted sutures are still placed in the area of the pouch shoulder to secure the telescope, and the pouch is repositioned stably against the abdominal wall

If sliding or prolapsing occurs chronically intermittently, inflammation and scarring may lead to morphologic destruction of the NV. There is an absolute indication for the reconstruction of the valve, usually from the afferent loop (Fig. 3).

#### Class 1d: additional scars and thickening or dilatation of the afferent loop

**Fig. 4:**
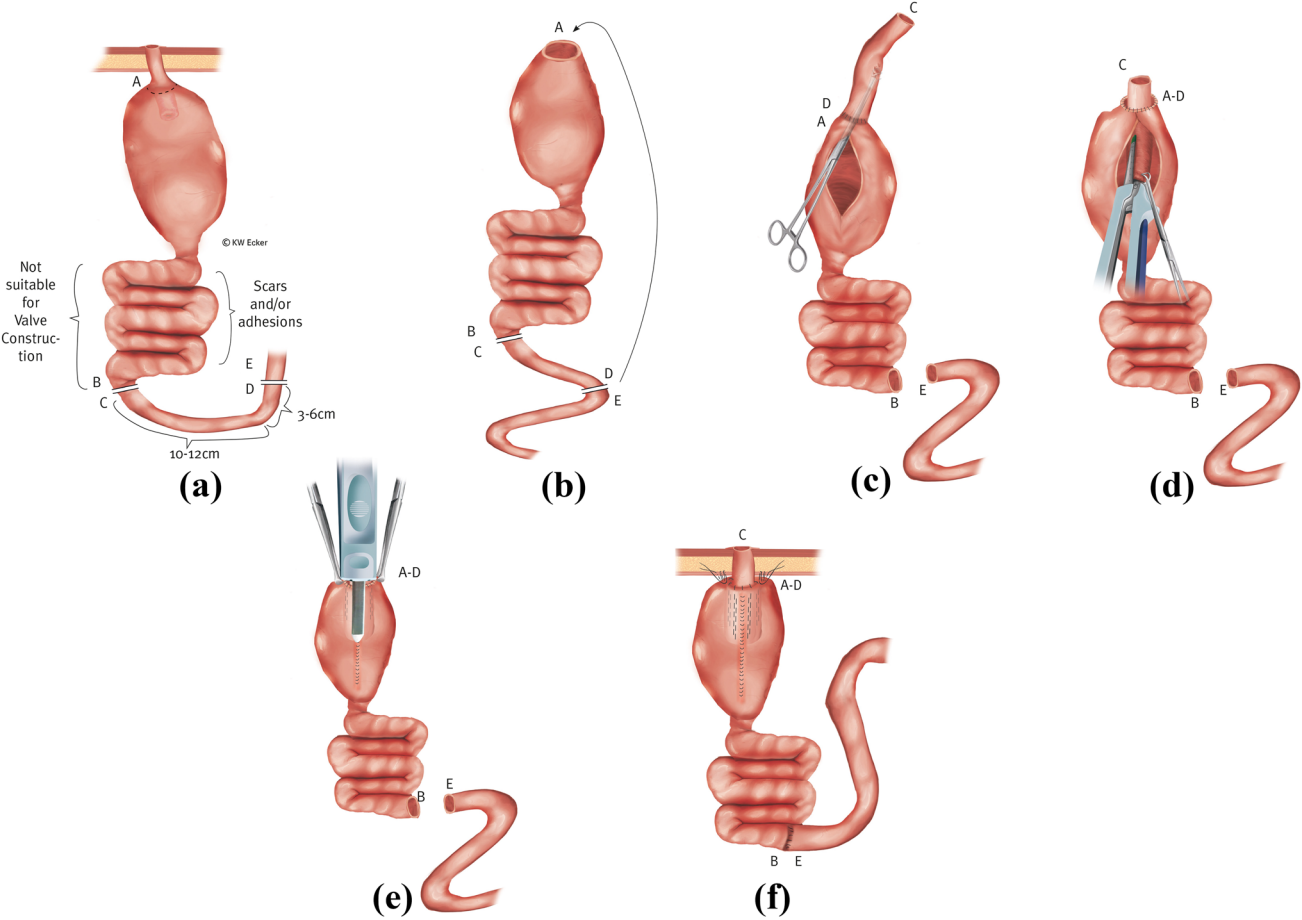
**Reconstruction of the nipple valve (NV) from a transposed higher loop (class 1d)**. (a) In case of a destroyed, i.e., no longer restabilizable, NV and an afferent loop unsuitable for NV formation, an orally located intestinal segment (C–D) of sufficient length is separated with preservation of the blood supply and prepared for transposition. (b) After resection of the old NV, the prepared intestinal segment is anastomosed with its oral end (D) to the former pouch outlet (A). (c) After completion of the enteroanastomosis (A–D), a Babcock clamp is inserted into the new outlet duct (the isoperistaltically transposed intestinal segment) via a longitudinal incision of the anterior wall to initiate intussusception. (d) As with an original construction, the NV is stabilized on both sides of the irradiating mesentery with one staple application on each side (four rows) using the bladeless linear cutter. (e) Across the front wall suture, the outer cuff of the NV is stapled against the pouch wall with the bladeless linear cutter. (f) After restoration of bowel continuity by enteroanastomosis (B–E), telescope securing sutures are applied to the pouch shoulder, and the pouch is repositioned with stable attachment to the abdominal wall.

In some unfavorable cases, the afferent loop is shown to be unsuitable for valve formation due to scarring and/or inflammatory changes. In cases where the loop can remain in situ with undisturbed function, it is advisable to construct a new NV from a transposed higher loop of the small intestine after resection of the old valve (Fig. 4). The same procedure is recommended if the afferent loop is considerably dilated.

### Pouch revision surgery (class 2)

Pouch complications are mainly manifestations of the underlying disease. While adenomas in familial adenomatous polyposis (FAP) can be resected endoscopically and pouchitis in ulcerative colitis should be treated conservatively or requires sacrifice of the pouch, fistulas may represent an indication for surgical revision. Three types are to be distinguished:

#### Class 2a: pouch–cutaneous fistulas

**Fig. 5:**
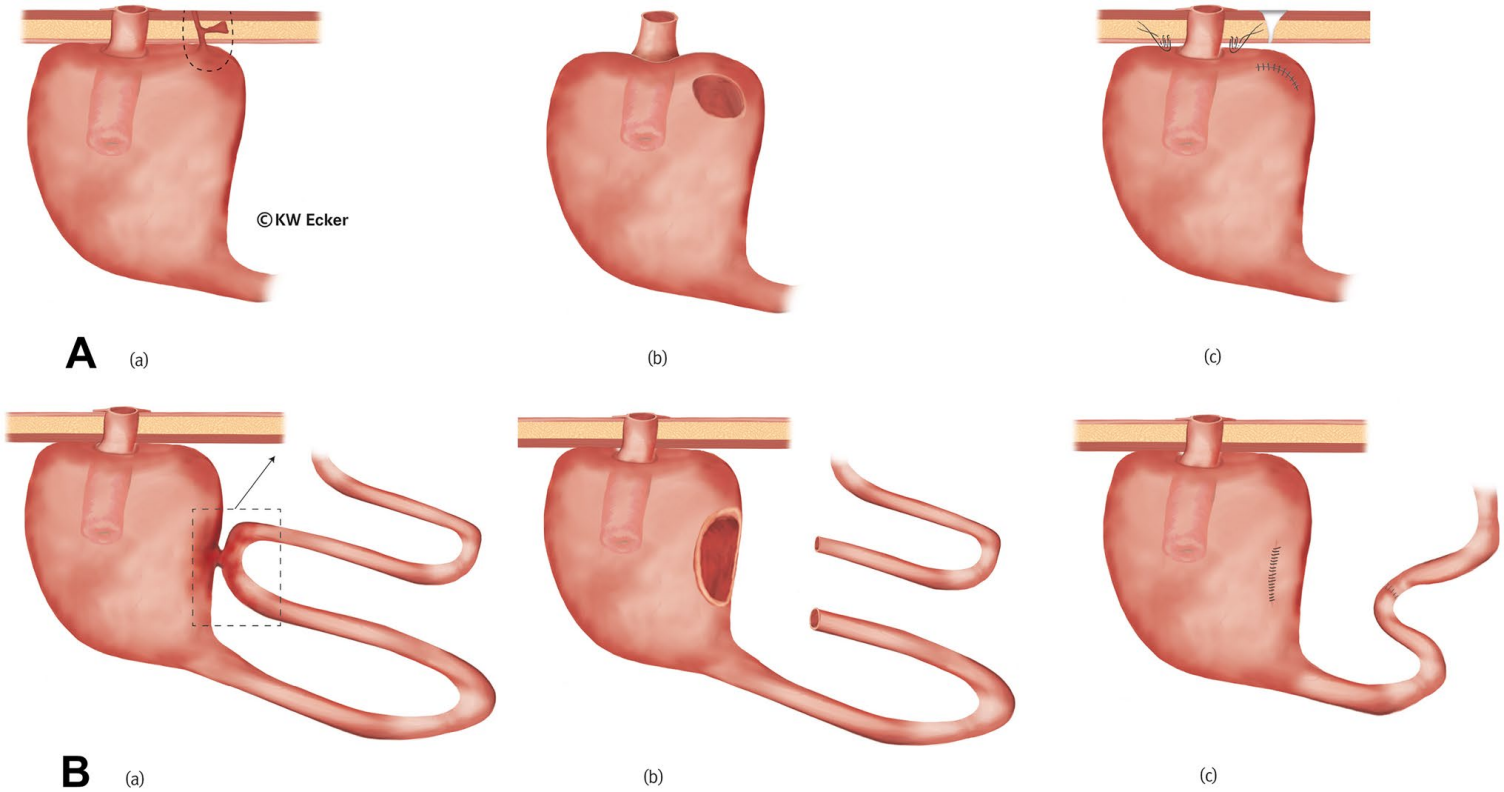
**Revisions for “simple” pouch fistulas**. **A** For pouch–cutaneous fistula (class 2a). (a) The fistula tract extends through the abdominal wall, with the external opening usually reaching the skin near the stoma. Occasionally, subcutaneous ramifications are found. (b) The fistula-bearing area of the pouch is completely excised. In favorable cases, the pouch can remain in situ after laparotomy, and in unfavorable cases, it seems better to remove it completely. (c) Fistula tracts in the subcutis are debrided as radically as possible. In case of pouch detachment, the pouch is stably repositioned after repair. **B** For pouch–enteral/vesical/genital fistula (class 2b). (a) A fistula exists between the pouch wall and an adherant ileal loop. The procedure is independent of whether the fistula originates in the pouch or in the intestinal loop. In the case of a pouch–vesical or pouch–genital fistula, the procedure is analogous. (b) The fistula area in the pouch wall is completely resected, as is the fistula-bearing intestinal segment. If the bladder and genitals (vagina) are involved, the procedure is analogous, but as organ sparing as possible. (c) The repair is completed with closure of the pouch, enteroanastomosis, or adequate care of the bladder or genitalia.

Pouch–cutaneous fistulas are usually an expression of a penetrating complication of a Crohn’s manifestation. In rare cases, they may also be “suture fistulas” resulting from the attachment of the pouch to the abdominal wall. Surgical repair is indicated depending on fistula productivity (Fig. 5A).

#### Class 2b: pouch–enteric/vesical fistulas

These fistulas are comparable to the usual enteric Crohn’s fistulas. The indication for surgical removal depends on the potential for sepsis (Fig. 5B).

#### Class 2c: fistulas surrounding the valve at the base

**Fig. 6:**
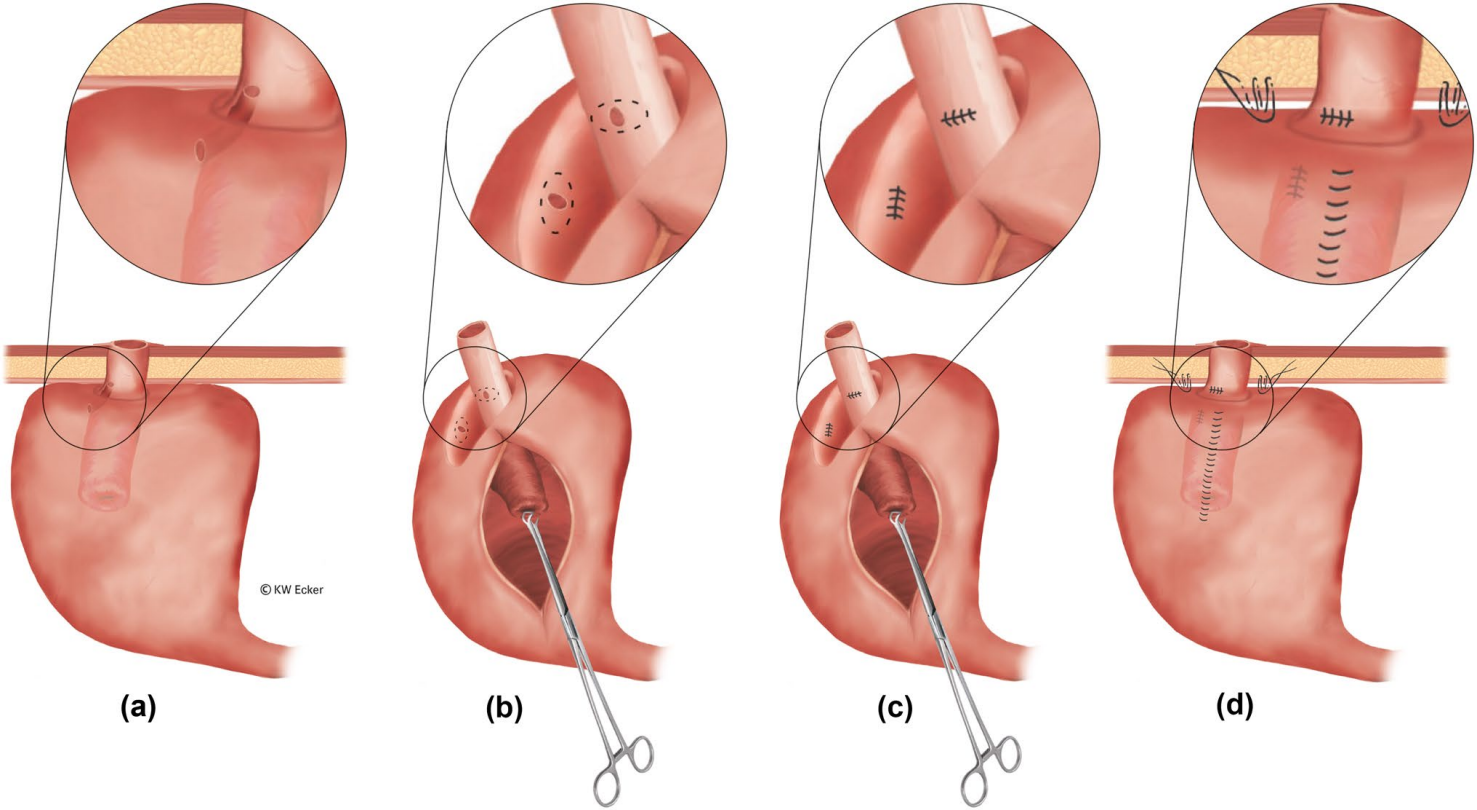
**Revision for “complicated” pouch fistula surrounding the base of the nipple valve (NV; class 2c)**. (**a**) There is a fistula between the pouch shoulder and the outlet duct surrounding the NV. (**b**) After complete mobilization of the pouch from the abdominal wall, the gap between the pouch and the outlet duct is carefully dissected, thus presenting both fistula openings preparatorily. It is helpful in this process if the pouch is opened at the anterior wall for better orientation. Thus, both fistula openings can be excised clearly. (**c**) Both fistula openings are closed by sutures. Care should be taken to ensure that when the gap between the pouch and the outlet duct is reclosed, these sutures do not come to rest on each other. (**d**) The closure sutures of the fistula openings are far apart. In this constellation, the pouch is securely anchored to the abdominal wall, and the stoma is reconstructed

These are the most difficult fistulas of all. Since they become symptomatic by partial or total incontinence, they counteract the objective of CI/KP and always represent an indication for treatment. Only if the pathogenesis is seen in Crohn’s disease can treatment with biologics be tried. However, subtle surgical closure is usually necessary (Fig. 6).

### Stoma revision surgery (class 3)

Special stoma complications in CI are related to the specific nature of the actual planar design at the skin level.

#### Class 3a: mucosal protrusion/full-wall prolapse

**Fig. 7:**
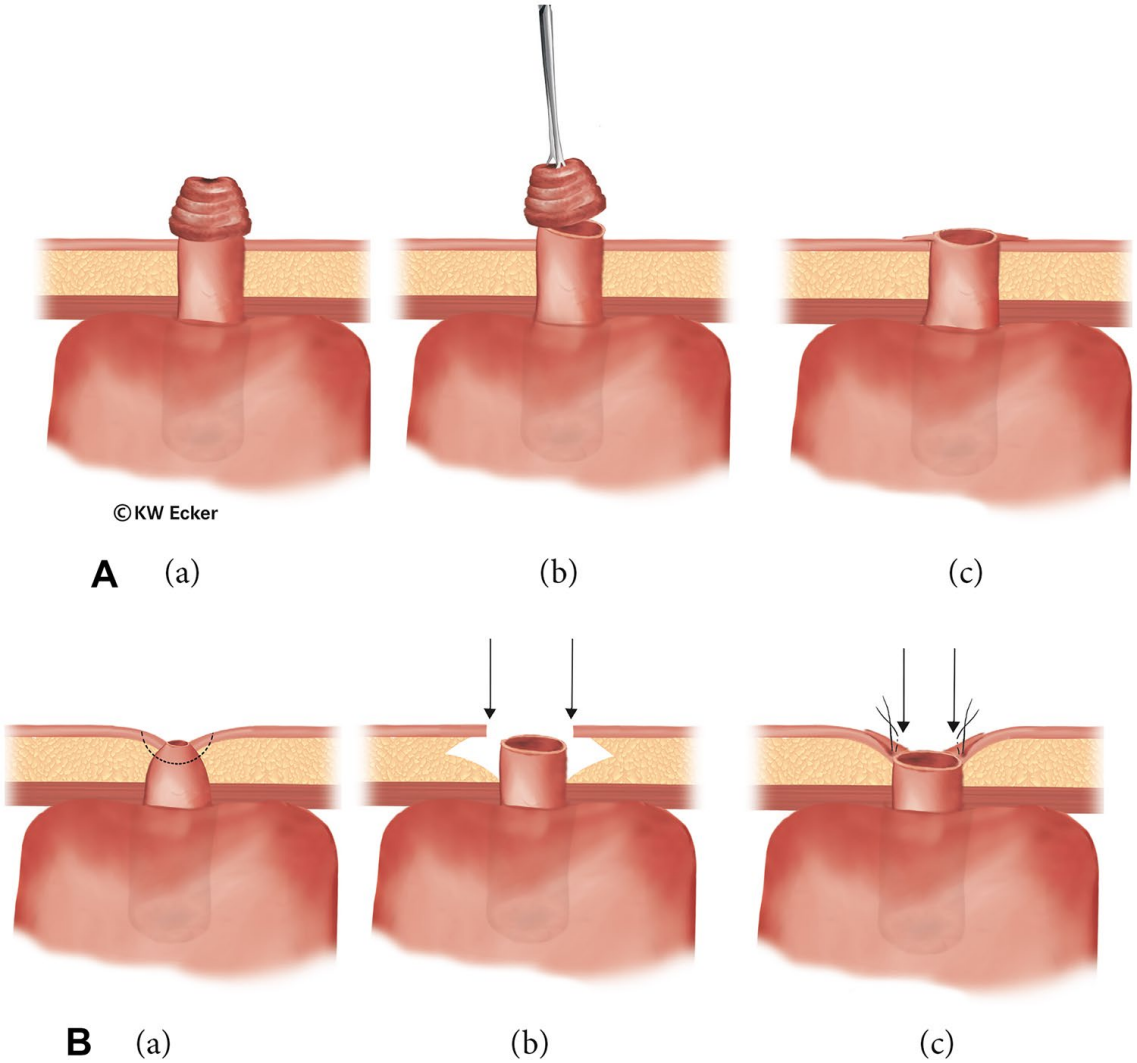
**Revision surgery for stoma complications**. **A** In case of mucosal protrusion (class 3a). (a) Even in cases where the stoma was primarily constructed flat, prolapse-like protrusion of the mucosa may occur in the long-term course, sometimes with formation of a small nipple as in a prominent ileostomy. Increased mucus production and difficult patch application represent indications for correction. (b) The small nipple is simply resected in front of the skin. (c) The bowel is again sewn flat into the skin. **B** In case of stenosis with/without stoma retraction (class 3b). (a) After local healing disturbances, stoma stenosis with/without retraction may occur. (b) In cases where a very voluminous subcutis is present, this is thinned out by circular excision of fatty tissue in order to be able to sew the bowel margin back in as a planar stoma without tension. (c) Although the newly created stoma lies in a depression of the skin and subcutis, it is no longer stenosed. Since no bag is necessary, the deeper position is irrelevant

In rare cases, mucosal protrusion may occur at the stoma. If the efferent loop was too generously dimensioned during stoma placement, full-wall prolapse of varying extent may also result. Depending on the excess mucosa, excessive mucus production is involved, which can overtax the absorption capacity of the special plaster for covering the stoma. Increased mucus secretion can first be managed conservatively using mini-bags; otherwise, surgical correction may be indicated (Fig. 7A).

#### Class 3b: stoma retraction with/without stenosis

If the efferent loop was inadvertently too short during stoma formation, or if the edge of the bowel was poorly perfused, retraction with/without stenosis may result. The consequences are more difficult intubation and usually also pain. Incipient stenosis at the skin level can usually be arrested with regular bougienage using Hegar’s pins. Sooner or later, however, corrective interventions are unavoidable (Fig. 7B).

### Reoperations at the afferent loop (class 4)

Revisions of the afferent loop are usually required because of stenosing or fistulating complications of Crohn’s disease.

#### Class 4a: afferent loop in S-pouch

**Fig. 8:**
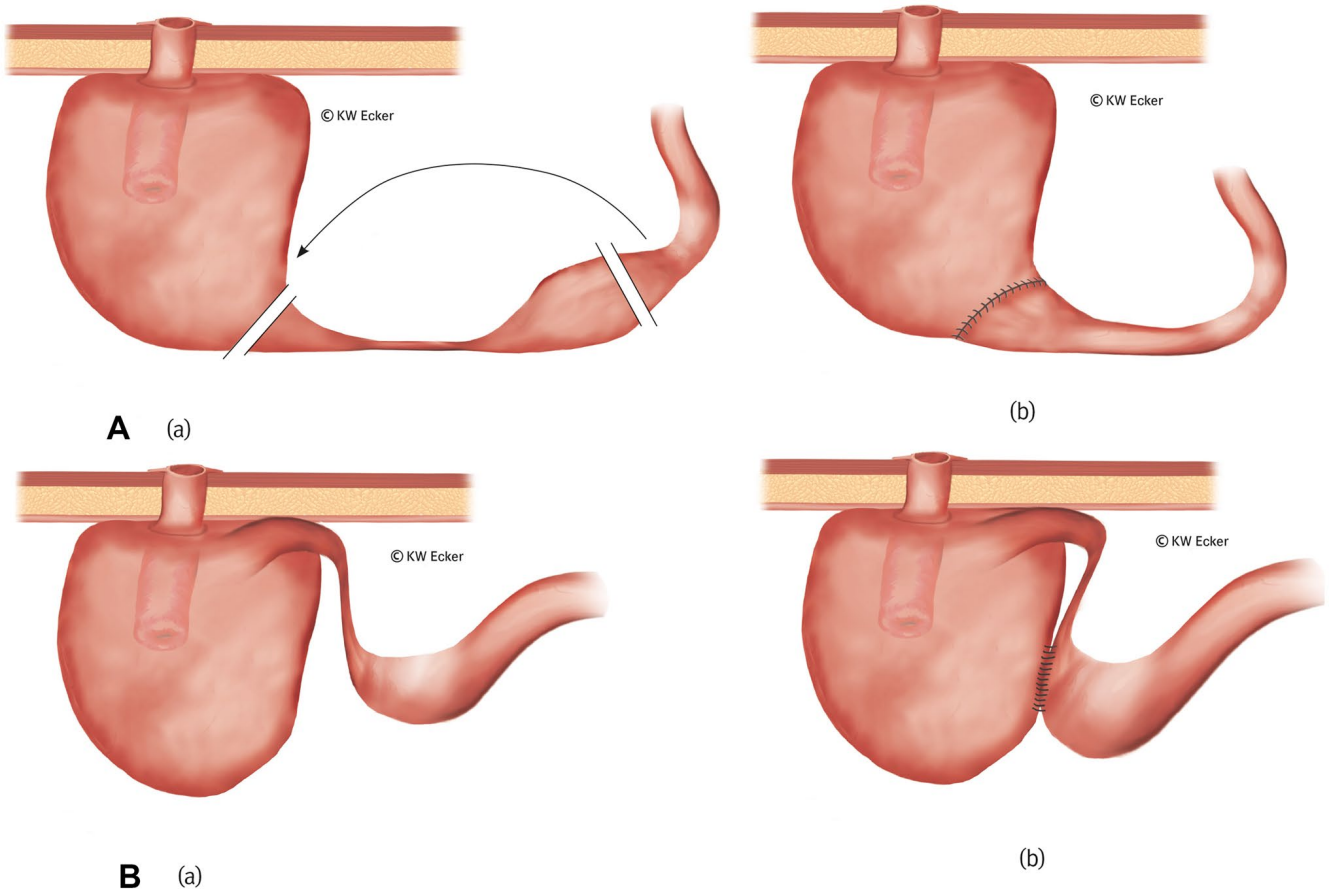
**Revision surgery for stenosis of the afferent loop**. **A** Ileal resection in case of an S-pouch (class 4a). (a) In an S-pouch, the afferent loop is located opposite the nipple valve (NV) and thus far away from the abdominal wall. It can usually be resected without the need to remove the pouch. (b) After resection, intestinal continuity is restored by enteroanastomosis between the oral ileum and the pouch. **B** Ileal bypass in case of a K pouch (class 4b). (a) In a K-pouch, the afferent loop is located close to the NV and therefore directly under the abdominal wall. Resection is difficult without complete mobilization of the pouch. In addition, there is potentially a risk of NV compromise involved in attempted resection. (b) A side-to-side anastomosis between the prestenotic ileum and the pouch in terms of an enteral bypass is simple to perform and largely eliminates unnecessary surgical risks.

In the case of an S-pouch, resection can be performed without releasing the pouch completely since the afferent loop is opposite the valve and easily accessible (Fig. 8A).

#### Class 4b: afferent loop in K-pouch

In the case of a K-pouch, resection of the afferent loop would require complete release of the pouch. In difficult situations, this could also endanger the NV due to its neighboring relationship to the afferent loop. Therefore, it may be wise to perform an intestinal bypass in selected cases (Fig. 8B).

## Discussion

While most surgical reports are limited to NV revision surgery [6], this paper presents a synoptic overview over the variety of correction methods. The broad technical repertoire of corrective surgery helps to achieve astonishingly high cumulative CI survival rates of up to 78.8% after 44 years in patients with at least one revision surgery [8]. A requirement for intervention may result from all structural components of the CI (NV, pouch, stoma, and afferent loop). Therefore, four revision classes are defined herein. There are large differences between these classes in terms of the influence of the primary surgical technique and the underlying disease on pathogenesis of the respective complications.

In agreement with the literature, complications of the NV (class 1) are purely technical and characterized by instability of the nipple formation. They may arise when the small intestine attempts to reverse the forced unnatural intussusception. Stabilization with metal staplers, as commonly performed today, has ameliorated this problem considerably but not eliminated it completely [1]. Surgical techniques correspond to the severity of the complication. This means that restabilization of an existing valve takes priority over constructing a new one. If in the latter case the afferent loop is suitable, the method is referred to as the “turnaround” procedure; if a transposed higher small bowel loop is necessary, this is referred to as “pedicle” repair [6].

In contrast, complications of the pouch (class 2) and the afferent loop (class 4) are clearly related to the underlying disease. Diffuse inflammation (pouchitis in ulcerative colitis) requires sacrifice of the CI in favor of IS if conservative measures fail. On the other hand, penetrating complications of circumscribed inflammation (fistulas in Crohn’s disease) may be treated by limited excision, as with fistulas of other pathogeneses. The aim is to preserve pouch and function. In familial polyposis, recurrent adenomas can usually be resected endoscopically, and desmoids in the mesentery may be suppressed pharmacologically. Stenoses and fistulas of the afferent loop are indicative of Crohn’s disease, even in cases where the previous diagnosis was different. Because pouch fistulas that surround the valve at the base open in the outlet and thereby produce incontinence, they have traditionally been classified as valve complications [6]. However, the valve is morphologically uninvolved and must not be repaired in the process of surgical fistula closure. Therefore, in the authors’ opinion, these fistulas are more correctly classified as pouch fistulas. While immediate postoperative occurrence is rather to be considered a result of disturbed blood circulation or a suture insufficiency, all fistulas in the long-term course correspond to complications of Crohn’s disease. Regardless of the time of occurrence, the surgical corrective procedures are identical. For all types of late fistula, there are also conservative options (e.g., biologics) that can be used to avoid surgical intervention, or after surgery as prophylaxis against relapse as in non-pouch surgery [9]. Some complications of the pouch, such as foreign body accumulation (e.g., enteroliths), can be treated endoscopically [10].

In stoma complications (class 3), technical inadequacies, connective tissue weakness, unusual aging processes, and disease recurrence may be involved in complication pathogenesis. In this sense, the focus in CI is particularly on mucosal protrusion and retraction. If local correction is indicated, it is often to be performed in conjunction with abdominal revision for other complications.

Revision surgery is without a doubt at least as demanding as primary CI/KP surgery. However, in skilled hands, it can be extremely successful and thus represents an integral part of pouch surgery. Without exaggerating, we would like to note that apart from severe pouchitis, there is hardly any complication in CI that could not be eliminated by revision surgery. This is not only important for restoring the best possible quality of life: of even greater importance seems to be the avoidance of pathophysiological consequences of resection of terminal ileum sections in terms of hardly compensable water and electrolyte losses. Interested surgeons are recommended and encouraged to participate in technical workshops and to perform their first procedures under the supervision of an experienced tutor.

## Conclusion

Based on classes of complications, the specialized surgeon may find differentiated techniques at their disposal to save the CI and avoid unnecessary sacrifice of the artificial continence organ. However, more reports are needed to evaluate the suitability and safety of these techniques from different perspectives.

## Data Availability

All data and materials used are secured digitally by the corresponding author.

## Code availability

No code available.
